# Re-rupture is not the only failure: tendon elongation and the 6-week nonoperative protocol for Achilles tendon rupture (Re: Happonen et al. Acta Orthop 2026; 97: 722-6. doi: 10.2340/17453674.2026.46872)

**DOI:** 10.2340/17453674.2026.46973

**Published:** 2026-10-19

**Authors:** Filippo Spiezia, Francesco Oliva, Nicola Maffulli

**Affiliations:** 1 Department of Health Sciences, University of Basilicata, Potenza, Italy; 2 Department of Orthopaedic and Trauma Surgery, Ospedale San Carlo, Potenza, Italy; 3 Department of Sports Traumatology, Università Telematica San Raffaele, Rome, Italy; 4 Department of Trauma and Orthopaedic Surgery, Faculty of Medicine and Psychology, University La Sapienza, Rome, Italy; 5 Centre for Sports and Exercise Medicine, Barts and the London School of Medicine and Dentistry, Mile End Hospital, Queen Mary University of London, London, UK; 6 School of Pharmacy and Bioengineering, Keele University School of Medicine, Stoke on Trent, UK

*Sir*,—We read with interest the article by Happonen et al. [1] comparing 8- and 6-week nonoperative protocols for acute Achilles tendon rupture in 479 patients. They concluded that the shorter strategy “may represent a safe and effective alternative.” We congratulate the authors for such an in-depth study. We believe however that the data supports a narrower conclusion.

The only endpoints were re-rupture requiring surgery and failed nonoperative management requiring surgery. Re-rupture is the most visible failure of a healing Achilles tendon, but not the only one. The healing tendon continues to elongate for 6 months, even after surgical repair, and full function takes at least 12 months to recover [2]. Elongation is accompanied by calf muscle atrophy and persisting plantarflexion strength deficit [3]. Without a measure of tendon length, such as the Achilles tendon resting angle [4], and without measurements of strength, patient-reported outcomes, or return to activity, the effectiveness of a shorter protocol cannot be fully evaluated. At 6 weeks, protection is withdrawn while the tendon is still elongating.

Overall failure did not actually decrease. Re-ruptures requiring surgery and failed nonoperative management together occurred in 8/251 (3.2%) patients in the 8-week group and 8/228 (3.5%) in the 6-week group, and failed nonoperative management rose from 0.4% to 1.8% (95% confidence interval −0.5 to 3.2 percentage points). With so few events and no prespecified non-inferiority margin, the investigation could not demonstrate equivalence: “comparable” means only that no difference was detected.

The 2 groups belong to consecutive periods, the later spanning the COVID-19 pandemic, when exposure to sport may have been reduced. Patients treated at other centers were not included, re-ruptures managed nonoperatively were not reported, and the duration of follow-up is not stated. Athletes were preferentially operated on, and operative management fell from 13% to 7.9%, leaving a nonoperative population with a mean age of about 50 years. The findings cannot be extrapolated to active, high-demand patients.

After percutaneous repair, slowing the progression of rehabilitation, with the same immediate weightbearing, produced less tendon elongation, a better Achilles Tendon Total Rupture Score, and greater symmetry of isometric strength and calf circumference at 12 months than a faster traditional protocol [5]. Following percutaneous suture, our patients now keep the boot until 10–12 weeks and introduce eccentric loading at about 12 weeks and plyometrics at 5 months [6]. A tendon healing without sutures has no mechanical safeguard against elongation and arguably needs at least as much protection. Structural indices do not always translate into patient-reported function [7]: this is exactly why both should be measured before a shorter protocol is endorsed.

Finally, the 14% complication rate after operative management, with 8% infections, is reported without the surgical technique. Minimally invasive repair offers function comparable to open repair with fewer complications [8]. This matters when operative and nonoperative options are weighed.

We would welcome the authors’ comments on whether tendon length or functional outcomes are available for a subset of their patients.

## References

[1] Happonen V, Koivisto A, Härkönen A, Kröger H. Nonoperative treatment of Achilles tendon rupture with 6- versus 8-week protocol: a single-center cohort study from Finland. Acta Orthop 2026; 97: 722-6. doi: 10.2340/17453674.2026.46872. PMC13624580 42812010

[2] Eliasson P, Agergaard A S, Couppé C, Svensson R, Hoeffner R, Warming S, et al. The ruptured Achilles tendon elongates for 6 months after surgical repair regardless of early or late weightbearing in combination with ankle mobilization: a randomized clinical trial. Am J Sports Med 2018; 46(10): 2492-502. doi: 10.1177/0363546518781826. 29965789

[3] Heikkinen J, Lantto I, Piilonen J, Flinkkilä T, Ohtonen P, Siira P, et al. Tendon length, calf muscle atrophy, and strength deficit after acute Achilles tendon rupture: long-term follow-up of patients in a previous study. J Bone Joint Surg Am 2017; 99(18): 1509-15. doi: 10.2106/JBJS.16.01491. 28926379

[4] Carmont M R, Grävare Silbernagel K, Brorsson A, Olsson N, Maffulli N, Karlsson J. The Achilles tendon resting angle as an indirect measure of Achilles tendon length following rupture, repair, and rehabilitation. Asia Pac J Sports Med Arthrosc Rehabil Technol 2015; 2(2): 49-55. doi: 10.1016/j.asmart.2014.12.002. PMC5730640 29264240

[5] Maffulli N, Gougoulias N, Maffulli G D, Oliva F, Migliorini F. Slowed-down rehabilitation following percutaneous repair of Achilles tendon rupture. Foot Ankle Int 2022; 43(2): 244-52. doi: 10.1177/10711007211038594. PMC8841642 34581220

[6] Maffulli N, Spiezia F, Migliorini F, Maffulli G, Oliva F. Walking on the Achilles tendons. Br Med Bull 2026; 157(1): ldag009. doi: 10.1093/bmb/ldag009. 41697883

[7] Maffulli N, Migliorini F, Oliva F, Maffulli G, Spiezia F. Percutaneous repair of acute Achilles tendon tears with minimally invasive augmentation with the tendon of flexor hallucis longus: a 5 years outcome study. Br Med Bull 2026; 159(1): ldag018. doi: 10.1093/bmb/ldag018. 42424592

[8] Feng S M, Maffulli N, Oliva F, Paul C, Fehske K, Plaass C, et al. Evidence-based clinical practice guidelines on the surgical management of acute Achilles tendon rupture. Br Med Bull 2026; 157(1): ldaf026. doi: 10.1093/bmb/ldaf026. 41528729

